# Resocializing Expertise: Becoming Ordinary in Extra-Ordinary Times

**DOI:** 10.1007/s11013-026-10029-w

**Published:** 2026-11-13

**Authors:** Hiroko Kumaki

**Affiliations:** Department of Anthropology, Oberlin College, Oberlin, USA

**Keywords:** Disaster, Mental healthcare, Expertise, Community care, Japan

## Abstract

The 2011 earthquake, tsunami, and nuclear disasters in Japan seemed an extra-ordinary set of circumstances that required highly specialized and scalable interventions. In response, disaster mental health professionals from across Japan were mobilized on an unprecedented scale. However, ethnographic research on disaster mental healthcare in the affected regions shows that rather than foregrounding their skills and training, practitioners emphasized the ordinary. Working outside the familiar social and institutional contexts that legitimized their practice, they re-embedded their training and practice within the ordinary social relationships of the communities and teams they served, resocializing their expertise. Becoming ordinary became a condition of possibility for care, enabling care providers to gain the trust of community members, provide support that was locally meaningful, and work effectively across professional boundaries. As crises become the new normal, medical and mental health training may involve attending to how expertise is resocialized within the ordinary social contexts in which the effects of disasters unfold and care takes place.

## Introduction

“We provide ordinary care as ordinary community members.” This was a refrain I heard from mental healthcare practitioners offering care in the communities affected by the 2011 earthquake, tsunami, and nuclear disasters in Japan (hereafter, the “2011 disaster”). As practitioners went into the disaster-affected communities, they learned to interact with community members not as professionals but as fellow residents—having tea, cooking, exercising, and even bathing with them. They dressed casually, in jeans and sweatshirts, often making themselves indistinguishable from residents.

Given the scale and combination of events that made the 2011 disaster a subject of world headlines, the emphasis on the ordinary stood out to me as I carried out ethnographic research in the affected areas. On March 11, 2011, a massive earthquake of magnitude 9.1 occurred off the coast of northeastern Japan, triggering tsunami waves that took nearly 20,000 lives, and devastated the infrastructure of the area. This included the nuclear reactors located in Fukushima, resulting in radioactive fallout and the evacuation of approximately 160,000 people at its height (Fukushima Prefecture, 2026). Why an emphasis on the ordinary in such extra-ordinary conditions?

In the wake of extra-ordinary events, such as large-scale disasters and wars, there is a prevailing sense that specialized intervention is needed to address the extent of the geographical, demographic, and economic damage incurred. One of the challenges of providing disaster mental healthcare in such conditions is to scale its expertise to meet the scale of the disaster (Dole, 2020, 2023). In fact, after the 2011 disaster, the Japanese government mobilized mental healthcare professionals from across Japan on an unprecedented scale, creating conditions in which scaling expertise seemed critical. Nevertheless, while practitioners were mobilized for their specialized credentials, my ethnography shows that effective care depended first on their ability to become ordinary.

Based on over a decade of ethnographic fieldwork in areas of northeastern Japan affected by the 2011 disaster, I examine how disaster mental healthcare professionals shaped care activities in ways that emphasized the “ordinary,” despite the seemingly extra-ordinary conditions they faced. My fieldwork took place mainly in Miyagi and Fukushima, both heavily affected by the 2011 disaster. I volunteered with and shadowed practitioners in organizations launched after the disaster to offer mental healthcare to affected communities. My main interlocutors were the practitioners themselves, including psychiatrists, psychologists, nurses, and social workers, who worked together in multidisciplinary teams.

Disaster mental health practices in areas affected by the 2011 disaster reveal a paradox: while practitioners were mobilized because of their training and specialized expertise, they often encountered situations in which their expertise was not immediately welcomed or useful to the communities they worked with. Christopher Dole (2020) describes the “work of therapeutic scalability” or the labor involved in extending psychiatric and psychological expertise across geographically dispersed populations of disaster survivors. My ethnography elucidates a related process in a different direction. Rather than prioritizing the implementation and extension of their therapeutic knowledge and practice, practitioners sought to establish an everyday presence by cultivating relationships and participating in ordinary activities that seemingly had little to do with their professional training. Emphasizing the “ordinary” became a pragmatic and ethical approach that shaped the possibilities of care.

Becoming ordinary describes how mental healthcare professionals learned to set aside their assumptions and frameworks for intervention shaped by their training. As practitioners entered the disaster-affected communities, they found themselves outside the social and institutional contexts that had legitimized their expertise and enabled their practice. This required them to find a place for themselves and their expertise in unfamiliar social settings. They invested much of their time and energy in ordinary activities before they could begin to enact, even partially, what they were trained to do. However, becoming ordinary did not entail abandoning their expertise. Instead, by cultivating trusting relationships with community members and the teams they were mobilized to work with, practitioners creatively reshaped and embedded their practice within the specific contexts in which they worked. I call this process the *resocialization of expertise*.

As a non-professional member of the multidisciplinary teams I worked with, I became implicated in the process of resocialization. I initially entered the field to understand how disaster mental healthcare operated as a response to disasters in Japan. Often called *kokoro no kea* or “care for the heart,” the practice had become a widespread and seemingly taken for granted response in Japan by the 2011 disaster. However, what practitioners did under its name remained diverse and difficult to pinpoint. I first reached out to established professional institutions and workshops focused on trauma care and training. However, I was often told I would need professional credentials to participate. In contrast, the community-based disaster mental healthcare organizations that provided care in the affected communities welcomed my participation precisely because they did not consider professional credentials as a prerequisite for contributing to their work.

Rather than working separately with practitioners from different professions, I worked with teams in which psychiatrists, psychologists, public health nurses, psychiatric nurses, social workers, and others engaged together in the everyday life of disaster-affected communities. As I discuss further below, the roles team members played were fluid rather than strictly determined by their professional training, and boundaries between experts and residents were not always clear-cut. This fluidity facilitated my participation in the teams’ activities, through which I became resocialized as a temporary resident and team member alongside practitioners. My own access to the field, therefore, was shaped by the very emphasis on becoming ordinary that I came to examine ethnographically.

In what follows, I begin by discussing the training and practice of expertise as a social process. The development of disaster mental healthcare expertise in Japan itself shows how this expertise was resocialized and transformed as it was introduced from the U.S. The subsequent three ethnographic examples from the 2011 disaster-affected areas show how practitioners resocialized their expertise outside their familiar institutional or clinical settings by becoming ordinary. These examples show how investment in ordinary relationships and care practices ultimately became the ground for practitioners to (1) become accepted in the communities they served, (2) provide support that responded to community needs, and (3) work together as members of a multidisciplinary team.

This complicates the prevailing assumptions in disaster mental healthcare and global health that it is beneficial to scale up and provide specialized interventions in response to large-scale crises. The way disaster mental healthcare developed in Japan shows practitioners’ everyday labor of resocializing expertise to be an unrecognized and undertheorized dimension of care work and professional training. As practitioners were mobilized, they recalibrated their practices, shifting their focus from self-evidently medical and psychiatric concerns to everyday concerns, even dressing and acting as fellow community members rather than experts in white coats (see Blumhagen, 1979). In the process, they loosened their hold on what counted as “appropriate” intervention for a trained professional expert.

For community-based disaster mental healthcare, care is premised on the trust and presence cultivated through ordinary settings and practices, rather than the display of specialized knowledge, skills, and authority. Instead of scaling up to the extra-ordinary through increased specialization, I argue that scaling down to the ordinary may be key to enabling practitioners to provide care in disaster-affected communities, ultimately allowing them to make the most of their specialized training and skills. Resocializing expertise by becoming ordinary thus becomes a condition of possibility for care. I conclude with the implications for community-based post-disaster care and training.

## Resocializing Disaster Mental Health Expertise

In a time characterized by increasing specialization and siloing of expertise, it is perhaps natural to expect the development and implementation of specialized responses to extra-ordinary situations like disasters. Disaster mental healthcare in Japan has indeed developed in ways that seek to accumulate and codify specialized knowledge so that it can be mobilized across disasters. Following the 2011 disaster, a national database was created to accumulate knowledge about disaster mental health responses and develop protocols for future disasters (MHLW 2014). Professional training of disaster mental healthcare in Japan has also often focused on clinical symptoms, trauma, and specialized responses to disasters. Psychiatric associations and medical schools across Japan have hosted workshops on post-traumatic stress disorder (PTSD) and disaster response (see NICSDMH 2026).

Such responses tend to understand expertise as something that can be possessed and mobilized across different contexts. Their focus is on acquiring the specialized knowledge and skills that experts can scale and implement regardless of the time and place of a disaster. This understanding reinforces the common image of experts as authority figures who provide specialized knowledge and skills in a particular field (see Wynne, 2006). However, the emphasis on the ordinary that I encountered in my fieldwork challenged this understanding of disaster mental health expertise as something extra-ordinary that experts possess and mobilize to address extra-ordinary conditions.^1^

Scholars have discussed expertise as something that is “enacted,” achieved, performed, and legitimized through ongoing social and institutional processes (Carr, 2010; Starr, 1982). In other words, instead of simply a possession, expertise is a social process that requires active shaping, legitimization, and adjustment in order to exist and be practiced. Disaster mental healthcare makes this particularly salient, as increasingly specialized knowledge and skills, developed and practiced within specific institutional contexts, need to be made useful outside those contexts. It is usually implemented after crises as a new practice and therefore an unfamiliar intervention for the affected communities. Practitioners also work outside the clinical and institutional settings that previously legitimized their training and skills and therefore must establish their own presence and practice in a new social context.

While the challenge of making expert practices meaningful and acceptable for the general public has been articulated (Fujigaki, 2016; Wynne, 1996), less discussed is the actual work that experts carry out in communities, outside the bounds of their professional settings, to find a place for and enact their expertise in the first place (see Brodwin, 2013; Dole, 2025; Livingston, 2012). Here, rather than treating this mainly as an epistemic issue of creating, transferring, or translating knowledge, I approach it as a relational issue. Through resocialization, expertise becomes re-shaped and re-embedded in new social relationships and contexts, enabling it to be practiced in diverse settings. This process helps explain the emphasis on the “ordinary” that I repeatedly encountered among disaster mental health professionals during my fieldwork. Through ordinary relationships and activities, practitioners re-examined and reshaped the assumptions and social worlds they carried with their professional training, learning how to enact their expertise in different relational contexts.

In fact, resocialization was integral to disaster mental healthcare in Japan from before the 2011 disaster. Disaster mental healthcare was introduced from the U.S. after the 1995 Kobe Earthquake and subsequently adapted and embedded in the Japanese context. Less than a week after the Kobe Earthquake, a major Japanese newspaper referred to the 1994 Northridge Earthquake that occurred near Los Angeles. It told the readers how almost 32 million dollars were allocated by the U.S. government to what the newspaper termed a “*Kokoro no Kea* (care for the heart) Project,” leading to a total of around one million counseling sessions provided to the survivors (Asahi Shimbun, 1995). In response to unprecedented calls for mental healthcare for survivors, but without a clear sense of what that entailed, psychiatrists in Kobe began providing emergency psychiatric care and moral support to the survivors (An, 2001).

Psychiatrists in Kobe turned to disaster mental health responses that had been carried out in San Francisco in 1989 and Los Angeles in 1994 after major earthquakes in those areas (Hyogo Institute 2004). Anthropologist Joshua Breslau discusses one such study trip to Los Angeles, during which participants learned about the concepts of Stages of Disaster, trauma, and debriefing (Breslau, 2000:184-187). Modeled on the U.S. system, the first *Kokoro no Kea* Centers were established across the city of Kobe and the larger Hyogo Prefecture. *Kokoro no Kea* Centers were initially built on the premise that long-term mental health responses, particularly for PTSD, would be necessary for affected residents. However, while trauma was the initial focus, the centers gradually developed to address issues specific to the circumstances of the region.

Resocialization of disaster mental health expertise from the U.S. context to Kobe led to deemphasizing acute trauma and clinical interventions and increasingly centering social isolation and socially isolated deaths in the community. At the time of the 1995 disaster, *kokoro no kea* was closely associated with PTSD (Fassin & Rechtman, 2009), reflected in the official English name of the *Kokoro no Kea* Center in Kobe, “Hyogo Institute for Traumatic Stress.” Nevertheless, as a new institution addressing a conceptually new issue, the staff members responded flexibly to changing and emerging needs on the ground (Hyogo Institute 2004, Kato, 2014). Over time, social isolation and socially isolated deaths became the main foci of intervention for *Kokoro no Kea* Centers in Kobe. This meant deemphasizing clinical trauma and interventions through counseling, and emphasizing community building and social engagements among survivors.

Popular discourse around *kokoro no kea* also became broader to encapsulate mental health in general. Prior to the Kobe earthquake, psychiatric practices focused on diagnosing and treating only severe mental health symptoms, such as schizophrenia, bipolar disorder, atypical psychosis, and major depression. These symptoms were highly stigmatized, often leading to seclusion of patients from society. Kobe Earthquake was one of the first times in which mental healthcare was called for as a disaster response and provided more generally in the community beyond major symptoms and diagnoses. In the years leading up to the 2011 disaster, *kokoro no kea* became a prevalent concept for addressing not only disasters but also everyday social and psychological distress (Kitanaka, 2012, MLHW 2011).

By the 2011 disaster, *kokoro no kea* had become an established response to disasters in Japan at the national and professional level. However, while community-based disaster mental healthcare increasingly emphasized the everyday social psychological distress of disaster survivors, the training of psychiatrists and psychologists remained focused on clinical symptoms and procedures. Thus the 2011 disaster created another moment of resocialization, as nationally mobilized practitioners now faced the challenge of bringing their expertise beyond the institutional settings in which they had been trained and practicing.

In the wake of the disaster, mental healthcare response took place at an unprecedented pace and scale. Within a few days of the disaster, the affected prefectures in northeastern Japan requested the Ministry of Health, Labour, and Welfare to send in teams of *Kokoro no Kea* providers to their region in anticipation of mental healthcare needs. Within a year of the disaster, 3,504 mental healthcare professionals from across Japan had entered the disaster-affected regions as ad hoc *Kokoro no Kea* teams, operating under the government mandate (MHLW 2012). *Kokoro no kea* teams usually consisted of multidisciplinary professionals, including psychiatrists, public health nurses, social workers, occupational therapists, pharmacists, clinical psychologists, and administrative staff. They were organized through networks of medical universities, medical associations, and hospitals across Japan and stayed in the region for a few days to, in some areas, more than a year. School counselors were also mobilized from the metropolitan areas in the immediate aftermath of the disaster. As *kokoro no kea* teams finished their activities, *Kokoro no Kea* Centers were established across the affected regions to provide long-term care. Psychiatry departments in local medical universities took on the role of carrying out research on mental health effects and advising the newly established *Kokoro no Kea* Centers.

As these institutions were primarily based in urban areas, *kokoro no kea* remained an unfamiliar practice in many rural communities of northeastern Japan where practitioners were mobilized. The emphasis on preventing social isolation and socially isolated deaths among survivors further required practitioners to work outside the clinical settings and engage with the everyday lives of disaster-affected communities. Unlike in clinical settings where professional roles and expectations were more clearly defined, practitioners often had to become acquainted with communities, cultivate social relationships, and find a place and role in the communities and teams they served. In order to create social belonging for survivors, practitioners themselves first had to socialize themselves into these communities through mundane activities that often fell outside their formal professional roles. The following three ethnographies show how practitioners resocialized their expertise by cultivating ordinary social relationships with disaster-affected communities and their own teams, striving first to become ordinary.

## Finding a Place in the Communities

“No *Kokoro no Kea* Allowed”

During fieldwork, I often heard stories about a sign posted at one of the evacuation shelters after the 2011 disaster that asked mental healthcare providers not to enter. While no one was able to tell me at which evacuation shelter this took place, this incident was brought up repeatedly by disaster mental healthcare providers. It was discussed as an example of the general sentiment among survivors who had grown tired of people coming to the shelters asking whether they were sleeping well, what their mood was, and whether they wanted to talk about their experience of the disaster. It was also brought up as an example of the difficulty of providing mental healthcare after disasters, when practitioners were often new to the communities they served and the need for psychological care was not immediately apparent.

As I visited *Kokoro no Kea* Centers and other organizations across northeastern Japan,^2^ conversations with practitioners often started with the challenge they faced in gaining acceptance from the local community. For example, in a tsunami-affected coastal town in Miyagi, the director of a social welfare organization reflected on the initial phase of their activities when the staff were outright rejected by community members. “When we launched this organization, our activities were not welcomed by the residents. Rejected and hurt, the staff member would come back to the office crying, telling me that they could no longer continue. I will never forget the fierce look one of them gave me when I told her to go back again the following day regardless.” Stories of staff members knocking on doors in tears and of residents annoyed by their daily visits were heard in disaster-affected sites in the first years after the 2011 disaster.

One of the main reasons for this difficulty was that disaster mental healthcare organizations were usually newly established after disasters and therefore unfamiliar to the disaster-affected communities. In addition, many of the communities in northeastern Japan devastated by the 2011 disaster were unfamiliar with psychological care itself. Thus, the experts and the organizations, as well as the expertise they were there to provide, were not yet embedded in the social and relational contexts of the region. As a result, care providers had to first find a place in the community where they worked, gradually establishing their presence. Instead of educating the community about mental health or the services they were there to offer, practitioners often did so by deemphasizing their credentials and first becoming part of the local ways of life and practices of managing health. In other words, they resocialized themselves and their practice within the existing relationships of affected communities.

For instance, rather than emphasizing their expert role, many guidelines came to recommend that care providers carry out *kokoro no kea* “in disguise.” Practitioners visited shelters without wearing name tags that identified them as *kokoro no kea* providers and brought blood pressure monitors to approach survivors through the more familiar daily practice of “measuring blood pressure” as a way of gauging one’s health (Cabinet Office, 2012). In both Miyagi and Fukushima, I often accompanied practitioners to tea gatherings and workshops where they wore jeans and T-shirts, measured participants’ blood pressure, and made small talk with them. Through these interactions, practitioners gradually became acquainted with disaster survivors, earned their trust, and carved out a role for themselves in the communities.

As practitioners found a place for themselves in these communities, what they practiced as *kokoro no kea* also changed shape. While disaster mental health practitioners were mobilized across the disaster-affected areas for their knowledge and skills, their encounters and relationships with community members reshaped what they practiced as *kokoro no kea*. This became evident in 2012, a year after the disaster, when psychiatrists, psychologists, and social workers who had participated in government-mandated *kokoro no kea* teams gathered for a reflective meeting in Tokyo and produced a report (see NICDMH 2012a, 2012b). Notably, participants struggled to reach a consensus on what *kokoro no kea* was or should be.

While they entered the disaster-affected region as *kokoro no kea* providers, they reported engaging in activities that they did not necessarily identify as mental healthcare. One reported, “No one would come talk to us as *kokoro no kea* providers, so we asked them if they needed help with anything, which worked well.” Another shared, “We did everything we were asked to do that mostly had nothing to do with *kokoro no kea*, including helping with the logistics of the town administration. If we enter the regions with a narrow definition of *kokoro no kea*, then we would risk limiting the services we could provide” (NICDMH 2012b). Participants concluded that avoiding a strict definition of *kokoro no kea* was important for gaining access to survivors and responding to the unfolding situation on the ground.

The practitioners’ recalibration of what *kokoro no kea* practice meant and looked like illuminates how they moved away from emphasizing and performing the specialized skills and knowledge they had been mobilized for. Instead, they spent considerable time and energy responding to what was asked of them and earning the trust of the communities they worked with. This meant that instead of diagnosing and providing counseling, they were sorting donated supplies, helping with logistics, cleaning shelters, playing with children, and doing whatever was needed, regardless of their credentials. Scholars have shown that mental healthcare resources can easily become stressors than support when expertise does not align with the needs in the community (Cuthbertson & Lai, 2025; Kato, 2014). By remaining flexible about what counted as disaster mental healthcare and responsive to needs as they unfolded, these practitioners became increasingly socialized and embedded in the ordinary lives of the disaster-affected communities they supported.

A similar dynamic continued in the longer-term *Kokoro no Kea* Centers newly established in the region after the emergency response teams left. As discussed in the opening, staff members of *Kokoro no Kea* Centers often wore casual clothes and interacted with residents as fellow community members in ways that did not easily identify them as mental healthcare professionals. Rather than emphasizing their specialized expertise, they harnessed the “ordinary” to resocialize their practice by finding a place in the community and earning the trust of its members. As I discuss below, in the long-term, this provided the grounds on which they could eventually make use of their trained knowledge and skills, if at all.

## Finding Meaningful Ways to Support Disaster-Affected Communities

Becoming ordinary did not mean that practitioners completely abandoned the expertise they had acquired through professional training. Instead, disaster mental healthcare became possible through the process of resocialization, often in unexpected ways: practitioners re-embedded themselves and their expertise in the new contexts in which they worked, creating possibilities for enacting their specialized knowledge and skills. Although this did not always happen, when it did, their expertise often took altered forms. In the short-term response, practitioners often engaged in activities that might not have been expected of mental healthcare professionals, such as sorting relief supplies and helping with everyday or administrative needs. In the longer term, as teams became established in their communities, they increasingly engaged in interventions that addressed concerns more readily legible as psychiatric symptoms. At the same time, much of their work continued to consist of mundane practices that were not necessarily identifiable as mental healthcare.

The social and relational contexts in which practitioners worked thus shaped how their knowledge and skills might be put into practice. For example, I worked extensively with a *Kokoro no Kea* Center in coastal Fukushima, whose multidisciplinary staff had extensive training in their respective fields. The team included a psychiatrist, clinical psychologist, public health nurse, psychiatric nurse, social worker, physical therapist, and administrative staff member. Some were local, while others were newcomers to the region after the disaster. Despite their distinct professional and geographical backgrounds, they often engaged in activities that seemed to have little to do with their training or skill sets. Morning exercise sessions and tea gatherings, for instance, were common activities in disaster-affected areas, recreating practices familiar to residents from before the disaster. Fukushima was no exception, and I joined staff members who took turns hosting these gatherings.

Other activities at the Center also showed how practitioners organized their daily practice around the needs of affected communities rather than their trained skills. In addition to tea gatherings and exercise sessions, they planted vegetables with residents, made recipe booklets featuring local food, and cooked what they harvested. They also hosted woodcarving workshops, hiking trips, and cooking classes for men with alcoholism, as well as held seasonal events and formed a rock band with younger unemployed adults. These activities brought together community members in ways that helped create a social safety net, preventing social isolation and socially isolated deaths, which had become major foci of intervention after the 1995 Kobe Earthquake (see Gagné, 2020; Kumaki, 2026). At the same time, organizing these activities allowed practitioners to become familiar with the ordinary life of the community, creating opportunities to make use of their training.

One of the more controversial activities that exemplified this process involved staff members organizing a public bath outing for a client who had been socially withdrawn and had not been taking care of himself. Instead of encouraging him to bathe at home, they planned a visit to a bathhouse as an unassuming way to get him out of the house and interact with others. On the day of the outing, a psychiatric nurse and a social worker drove the client to a local bathhouse. There, they encouraged him to buy his own ticket from the vending machine and say hello to the receptionist. In the bath, the client and staff members washed each other’s backs, soaked in the hot bath, and chatted with other visitors.

In the process, the nurse and social worker each put their training into practice. The nurse observed the client’s body and behavior as they bathed, looking for any physical or psychological symptoms that might need attention. As they soaked in the bath and later drank milk together – a common practice at bathhouses in Japan – the social worker casually asked the client about his life, his family, and his former job. This gave the staff members an opportunity to understand who he was, how he had become socially withdrawn, and what kinds of support he might need moving forward. Thus, rather than abandoning their expertise, practitioners re-embedded it in ways that integrated more seamlessly into the everyday lives of residents, making their distinct knowledge and training useful in unexpected ways.

Moreover, this approach allowed for more creative and playful engagements that fostered a sense of connection with the client and among practitioners. Going to a public bath and being naked together can be understood as a practice that leads to closer social relationships – or what is called *hadaka no tsukiai*, or “naked relationship,” in Japanese. By participating in this ordinary social activity, practitioners were not only attending to the client’s hygiene and assessing his condition, but also creating an approachable opportunity for a socially isolated client to form relationships with others. While some have criticized the public bath outing for crossing the boundaries of professional practice, this activity illustrates a form of care that might not have been possible or imaginable had practitioners insisted on interventions defined by their professional training and protocols.

Grounding their practice in the everyday life of the region not only reshaped how practitioners used their expertise but also broadened the concerns they responded to. Over the years, their work came to address not only the aftermath of the disaster, but also chronic social issues exacerbated by it. Veena Das (2006) has shown through her ethnography of violent events in India how communities affected by crises often reintegrate and manage pain through their daily routines. Unassuming everyday contexts, such as tea gatherings and bathhouse visits, offered opportunities for practitioners to support disaster-affected residents in living with and addressing the pain of the disaster in ways that clinically based, diagnosis-centered treatments might not.

At the same time, as scholars of disasters have shown, crisis response often draws attention and resources to chronic structural issues that were already present but became intensified and more visible in the wake of the crisis (Farmer, 2003; Oliver-Smith & Hoffman, 1999; Seale-Feldman, 2020). The activities of the Center in Fukushima reflected both the effects of the disaster and evacuation as well as long-standing social issues in the region. For example, activities for socially isolated elderly residents reflected the region’s aging population and the outmigration of younger generations. The focus on men with alcoholism and younger adults without jobs also reflected the limited employment opportunities in the rural region.

As they engaged with changing and emerging needs, mental healthcare practitioners increasingly came to understand their activities as filling gaps in existing medical and social welfare systems. For example, the director of the Center in Fukushima described a new group of clients they worked with, many of them young adults, who had moved to the region for decontamination work and stayed on. Many had fallen into poverty and were living as newcomers without family or acquaintances nearby to support them. Like the other groups of residents supported by the Center, these clients often had no specific diagnoses or conditions that connected them to medical or social welfare systems. Nevertheless, they bore substantial mental health burdens related to both the disaster and the social and economic structures that made them vulnerable. As a newly established organization after the disaster that gradually became part of the community, the Center came to address needs that had fallen through the cracks of existing systems.

The director described this work as a “stop-gap industry” (*sukima sangyō*), addressing issues that could not be neatly categorized as medical or psychiatric but nevertheless needed attention and care. By resocializing their expertise within ordinary community relationships, practitioners were able to recognize and respond to needs that fell outside formally designated professional roles and institutional responsibilities of existing medical, psychiatric, and social welfare services. The director’s characterization of this work as an “industry” underscores the extent of this underrecognized labor and the critical role it has come to play in post-disaster care. Ordinary social contexts therefore became important sites for addressing both the ongoing effects of the disaster and the chronic structural vulnerabilities amplified by it, particularly those overlooked by existing systems of care.

## Finding a Place in Multidisciplinary Teams

Resocializing their expertise in the ordinary proved useful not only for gaining acceptance from community members and providing meaningful care, but also for working effectively across professional boundaries. Disaster mental healthcare in Japan has been characterized by the multidisciplinary nature of its teams. In clinical settings, there is often a division of labor among practitioners, in which physicians, nurses, and social workers take on specific professional roles. In contrast, disaster mental healthcare practitioners in Japan work in multidisciplinary teams without a strict division of labor, often carrying out activities and roles that do not necessarily draw on their specialized training. Learning to work together across differences in training and professional expectations thus became key to functioning successfully as a team.

Basing their practice on mundane activities allowed practitioners to begin building relationships with one another. For example, a public health nurse, a clinical psychologist, and a psychiatrist might organize and attend a tea gathering, sitting around the table and chatting with residents. For a public health nurse, physically reaching out to the community was a familiar practice. For a clinical psychologist or psychiatrist, however, this could be unfamiliar territory. Working together to set up and clean up the events, talk with residents, and learn about the changing situation after the disaster allowed them to step outside of their usual professional roles and become acquainted with one another.

Such collaboration among different professionals, however, was not always easy. The expertise each professional was trained in often shaped how they identified relevant issues and what course of action they considered appropriate (Collins & Evans, 2008). Working together meant learning to work across differences in training, sometimes going against familiar ways of understanding problems and intervening in them. For practitioners, letting go of their hard-earned tools, even temporarily, could be experienced as destabilizing and, at times, threatening.

A clinical psychologist I met in a tsunami-affected town in Miyagi reflected on how disaster intervention challenged her expert identity. As a clinical psychologist, she valued setting clear boundaries in spatial, temporal, and relational terms, carrying out her practice in an office where those boundaries were clearly delineated. As she began working in a tsunami-affected town, however, she had to become accustomed to interacting with community members and other practitioners in everyday settings, under a canopy tent or in temporary homes, without clear spatial, temporal, or professional boundaries.

In the initial phase, differences in credentials and approaches created a sense of discouragement and even hostility among team members. The director of the Center in Fukushima recalled the days when the multidisciplinary staff members would not listen to him or to one another during meetings. Each morning, they had a meeting to go over the previous day and make plans for the day ahead. Yet, while gathered around the table, many staff members would sit absentmindedly looking at their phones. He showed me a photo of a card holder on the table with a note that read “Please look here,” asking participants to look up from their phones. “It was this bad at first,” he reflected with a laugh. “We had to learn to understand and accept each other’s ways.” It took them years of trial and error, along with considerable staff turnovers, before they were able to create a team that worked toward a common goal and saw their differences as assets rather than obstacles to care.

These tensions were even more salient when practitioners from outside the region came to provide support. Local practitioners were often suspicious of outsiders who had more educational and professional credentials and sometimes criticized local practices based on expectations that did not match the resources and conditions on the ground. The director of a mental healthcare organization in Miyagi noted that his first task was to repair the relationship between the organization and local public health practitioners. The previous director had insisted on particular ways of practicing mental healthcare and openly criticized local practices that did not match his expectations, leading local practitioners to refuse to work with him and the organization. Both directors had come from outside the region with degrees in clinical psychology, yet they approached their roles differently. The new director began by building relationships with the community. Instead of imposing his own professional expectations, he engaged with local practitioners in their everyday settings, participating in community events and organizing social gatherings to learn where the organization might be of assistance.

As practitioners learned to work together, resocialization in multidisciplinary teams did not require them to permanently set aside their specialized skills. Instead, they sought appropriate contexts and ways to put their training into practice. At the Center in Fukushima, a clinical psychologist from outside the region described her effort to secure an office space for her practice. The organization and its multidisciplinary team valued openness and collaboration. The spatial organization of the Center reflected this approach, as consultations initially took place in spaces visible and audible to every team member on the floor, often not as formal “consultations” but as part of group gatherings or seasonal events. It took the clinical psychologist three years of working with the team before she felt the time was right to convince her colleagues of the value of a separate office space where she could close the door and create a bounded space for her practice.

During my fieldwork, six years after the disaster, I was able to see the results of practitioners’ efforts to make multidisciplinary collaboration work. For example, the Center in Fukushima held monthly meetings in which all staff members, including psychiatrists, clinical psychologists, social workers, public health nurses, nurse practitioners, physical therapists, and guests like myself, gathered to discuss a case they were working on. The meeting consisted of respectful exchanges in which each participant offered a perspective on the case and was encouraged to ask questions and offer suggestions based on their expertise and experience. Even as a non-professional participant, I was also asked to contribute my thoughts and questions.

In one case of an adult *hikikomori*, or a client who had been withdrawn at home for years, the clinical psychologist discussed the mother-child relationship and attachment, the public health nurse discussed outreach efforts to keep the person socially engaged in the community, the social worker suggested daytime care activities and potential job opportunities, and the psychiatrist discussed diagnosis and medication. Bringing together this array of perspectives and suggestions allowed the team to provide more comprehensive support for the client. While the meetings were often long and intense, the willingness of each practitioner to sit together and learn from one another showed the potential of such collaboration across disciplinary boundaries. Reaching this stage, however, first required them to build rapport through mundane activities and resocialize themselves and their expertise within the team.

## Discussion

Ethnography of disaster mental healthcare after the 2011 disaster in Japan shows the limits of approaching expertise primarily as a possession of specialized skills that can be readily scaled across contexts. While practitioners were mobilized for their credentials, their ability to put their skills into practice depended on the relationships they cultivated in the communities and teams they served. Expertise became resocialized through a relational process in which specialized knowledge and skills became socially embedded, reshaped, and made useful in the contexts in which they were enacted.

Understanding expertise as a relational process draws attention to everyday relationships and practices of care that precede and sustain professional intervention. It shows that professional interventions must first enter and become embedded in existing social relationships and structures of care, rather than existing on their own or replacing them. For practitioners, this meant shifting their attention from demonstrating their specialized skills and credentials to cultivating relationships that could sustain their presence and give meaning to their practice within the particular communities and teams they served. While practitioners often found it unsettling to move beyond familiar professional protocols and practices, they gradually came to recognize mundane activities as crucial to fostering trust and creating the conditions in which their training could become useful.

It is important to note, however, that becoming ordinary did not entail a complete absence or rejection of the specialized knowledge and skills that practitioners acquired through their training. Instead, it entailed a deliberate reorientation of what their expertise could look like and how and when it might be put into practice. Rather than asserting their credentials and protocols at the outset, practitioners held them in reserve and recontextualized them, drawing on them later at an appropriate time and shape. In some cases, specialized practices reemerged in recognizable forms. In others, they took new forms as they became incorporated into ordinary activities that no longer appeared explicitly medical or psychiatric. Expertise was therefore reshaped and exercised selectively and situationally through ongoing relationships and negotiations at the site of practice, rather than determined in advance by professional training or institutional mandates.

Furthermore, resocializing expertise reshaped not only how practitioners provided care, but also what came to be recognized as requiring care. By keeping the definition of care and their roles open and becoming familiar with the everyday life of the community, disaster mental healthcare practitioners were able to identify and respond to the needs that fell outside existing medical and social welfare systems. Their activities came to address chronic structural issues that became more salient after the disaster, such as social isolation, chronic unemployment, precarious labor, and poverty that did not neatly align with psychiatric diagnoses or categories of public assistance. Practitioners came to inhabit a liminal space, functioning as what one interlocutor called a “stop-gap industry.” The seeming ordinariness of their activities, such as holding tea gatherings, exercising together, sharing meals, and bathing together enabled forms of care that addressed post-disaster suffering as lived, relational, and structural, rather than as explicitly clinical or bounded by the disaster itself.

As crises become the new normal, medical and mental health training may involve learning how expertise is resocialized within the ordinary social contexts in which the effects of disasters unfold and care takes place. During my fieldwork, medical and nursing students came to train at the disaster mental health organizations where I worked to experience community-based care and multidisciplinary teamwork. I observed how disorienting these experiences could be for students whose training emphasized increasingly specialized forms of professional practice and the demonstration of their competence (see Haas & Shaffir, 1977). Upon arrival, practitioners encouraged them to first set aside their skills and credentials and engage in mundane activities as fellow residents and colleagues. These experiences provided students with opportunities to contextualize and recalibrate their training through relationships with community members and their teams, and to understand their expertise as something continually shaped through those relationships.

Community engagement has been central to training medical professionals in cultural and structural competency (Davenport, 2000; Kleinman & Benson, 2006; Metzl & Hansen, 2014). In Japan, particularly after the 2011 disaster, medical education has increasingly incorporated community-based experiences. Yet, such approaches can inadvertently assume that practitioners’ knowledge and skills remain largely intact while being applied with greater sensitivity to local contexts. The ethnographic accounts presented here suggest a different orientation to community engagement and professional training. Beyond supplementing expertise with more sensitivity or local knowledge, expertise itself may need to be recalibrated through engagement with the ordinary relationships and practices of the communities and teams in which it is enacted. Resocialization thus becomes a generative process through which professional expertise is reshaped and meaningful care becomes possible.

## Conclusion

In a time when extraordinary conditions have become ordinary, many systems of intervention remain organized around increasing specialization and rigid professional boundaries. As a result, people and problems that do not fit within existing structures can fall out of reach, and collaboration across professional boundaries becomes even more difficult. Disaster mental healthcare in Japan, with practitioners’ emphasis on the ordinary, offers a potential alternative to this trajectory. In the wake of large-scale disasters, there is often an impulse to provide immediate, scalable, and specialized interventions. Nevertheless, my ethnography shows that effective care may also require practitioners to scale down to ordinary relationships and practices. Being present with others and adapting without immediately intervening may not be a retreat from expertise but a condition for its ethical and effective use. Medical and mental health training may therefore place greater emphasis not only on acquiring specialized skills but also on learning when and how to make use of them in everyday contexts, even when doing so requires substantial recalibration of professional expectations and practices. In prolonged crises, the ability to resocialize expertise through ordinary social relationships may be vital to responding to changing everyday needs and structural conditions.

## Data Availability

No datasets were generated or analysed during the current study.

## Footnotes

1 This emphasis on ordinariness in disaster mental healthcare can be situated within a historically specific context in which the authority of scientific expertise was increasingly contested following the 2011 disaster (Kobayashi, 2016; Shimazono et al., 2016). It also reflects longer-standing efforts to reform institutional psychiatry and develop alternative forms of mental healthcare in Japan (Kitanaka, 2012).

2 I discuss multiple *Kokoro no Kea* teams and *Kokoro no Kea* Centers throughout this article. While I identify their prefectural locations, I do not specify individual teams or Centers to protect the anonymity of my interlocutors. I also identify practitioners primarily by their professional roles to highlight how these shaped, but did not determine, the ways they resocialized their expertise in community-based care.

## References

[CR1] An, K. (2001). *Kokoro no Kizu o Iyasu to Iukoto*. Kadokawa Shoten.

[CR35] Asahi Shimbun. (1995). Kokoro no Tachinaori, Sasaetai Rinshoshinrishi ga Muryo Denwa Sodan Hanshin Daishinsai. *Asahi Shimbun*, January 23.

[CR2] Blumhagen, D. W. (1979). The doctor’s white coat. The image of the physician in modern America. *Annals of Internal Medicine,**91*(1), 111–116. 10.7326/0003-4819-91-1-111 88917

[CR3] Breslau, J. (2000). Globalizing disaster trauma: Psychiatry, science, and culture after the Kobe earthquake. *Ethos,**28*(2), 174–197. 10.1525/eth.2000.28.2.174

[CR4] Brodwin, P. (2013). *Everyday ethics: Voices from the front line of community psychiatry*. University of California Press.

[CR5] Carr, E. S. (2010). Enactments of expertise. *Annual Review of Anthropology,**39*, 17–32. 10.1146/annurev.anthro.012809.104948. Volume 39, 2010.

[CR31] Cabinet Office. (2012). *Hisaisha Kokoro no Kea Todofuken Taio Gaidorain*. Cabinet Office. https://www.bousai.go.jp/taisaku/hisaisyagyousei/pdf/kokoro.pdf

[CR8] Collins, H., & Evans, R. (2008). *Rethinking expertise*. University of Chicago Press.

[CR10] Cuthbertson, C., & Lai, J. (2025). When resources become stressors: Dynamics of the stress process in the Flint water crisis. *Culture, Medicine and Psychiatry,**49*(3), 544–560. 10.1007/s11013-024-09887-z 39775399

[CR11] Das, V. (2006). *Life and words: Violence and the descent into the ordinary* (1 edition). University of California Press.

[CR12] Davenport, B. A. (2000). Witnessing and the medical gaze: How medical students learn to see at a free clinic for the homeless. *Medical Anthropology Quarterly,**14*(3), 310–327. 10.1525/maq.2000.14.3.310 11036581

[CR13] Dole, C. (2020). Experiments in scale: Humanitarian psychiatry in post-disaster Turkey. *Medical Anthropology: Cross-Cultural Studies in Health and Illness,**39*(5), 398–412. 10.1080/01459740.2020.1755284 32394758

[CR14] Dole, C. (2023). Psychiatry, disaster, security: Mediterranean assemblages. *Culture, Medicine and Psychiatry,**47*(1), 62–81. 10.1007/s11013-022-09799-w 35930102

[CR15] Dole, C. (2025). *Living on: Psychiatry and the future of disaster in Turkey*. Stanford University Press.

[CR16] Farmer, P. (2003). *Pathologies of power: Health, human rights, and the new war on the poor*. University of California Press.

[CR17] Fassin, D., & Rechtman, R. (2009). *The empire of trauma: An inquiry into the condition of victimhood*. Princeton University Press.

[CR33] Fukushima Prefecture. (2026). *Transition of the Number of Evacuees—Fukushima Revitalization Information Portal Website*. https://www.pref.fukushima.lg.jp/site/portal-english/en-1-4-1.html

[CR18] Fujigaki, Y. (2016). Gakusha to Shite no Sekinin to STS. *Journal of Science and Technology Studies,**12*, 157–166.

[CR19] Gagné, I. (2020). Dislocation, social isolation, and the politics of recovery in post-disaster Japan. *Transcultural Psychiatry,**57*(5), 710–723. 10.1177/1363461520920348 PMC7583447 32903157

[CR20] Haas, J., & Shaffir, W. (1977). The professionalization of medical students: Developing competence and a cloak of competence. *Symbolic Interaction,**1*(1), 71–88. 10.1525/si.1977.1.1.71

[CR21] Hyogo Institute for Traumatic Stress. (2004). Hanshin Awaji Daishinsai to Kokoro no Kea. *Hyogo Institute for Traumatic Stress*. http://www.j-hits.org/hanshin_awaji/kokoro_h_a/index.html

[CR22] Kato, H. (2014). Kokoro no Kea no Arikata o Megutte. *Fukko (10 Go)*, *6*(1), 60–65.

[CR23] Kitanaka, J. (2012). *Depression in Japan: Psychiatric cures for a society in distress*. Princeton University Press.

[CR24] Kleinman, A., & Benson, P. (2006). Anthropology in the clinic: The problem of cultural competency and how to fix it. *PLoS Medicine*. 10.1371/journal.pmed.0030294 PMC1621088 17076546

[CR40] Kobayashi, T. (2016). Motto Mae Kara Manande Oku Beki Datta Koto. *Kagaku Fushin no Jidai o Tou: Fukushima Genpatsu Saigai go no Kagaku to Shakai *(pp. 8–29). Godo Shuppan.

[CR39] Kumaki, H. (2026). Living a “good death”: Caring for solitary deaths in Japan. *Medical Anthropology Quarterly,**40*(1). 10.1111/maq.70051 41468357

[CR28] Livingston, J. (2012). *Improvising medicine: An African oncology ward in an emerging cancer epidemic*. Duke University Press. 10.1080/13648470.2014.929634 24962205

[CR25] Ministry of Health, Labour and Welfare (MHLW). (2011). *Minna no Mentaru Herusu*. http://www.mhlw.go.jp/kokoro/.

[CR26] Ministry of Health, Labour and Welfare (MHLW). (2012). *Higashi Nihon Daishinsai no Hisaichi ni Haken Sareta Kokoro no Kea Chiimu ni Tsuite.* https://www.mhlw.go.jp/seisakunitsuite/bunya/hukushi_kaigo/shougaishahukushi/kokoro/shinsai/

[CR27] Ministry of Health, Labour and Welfare (MHLW). (2014). *Saigai Haken Seishin Iryo Chiimu (DPAT) Katsudo Youkou*. https://www.mhlw.go.jp/seisakunitsuite/bunya/hukushi_kaigo/shougaishahukushi/kokoro/ptsd/dpat_130410.html

[CR29] Metzl, J. M., & Hansen, H. (2014). Structural competency: Theorizing a new medical engagement with stigma and inequality. *Social Science & Medicine,**103*, 126–133. 10.1016/j.socscimed.2013.06.032 PMC4269606 24507917

[CR30] National Information Center of Disaster Mental Health (NICDMH). (2012a). *“Higashi Nihon Daishinsai Kokoro no Kea Katsudo ni Kakaru Iken Koukan Kai” Houkoku*.

[CR6] National Information Center of Disaster Mental Health (NICDMH). (2012b). *Higashi Nihon Daishinsai Kokoro no Kea Chiimu Haken ni Kansuru Chousa Houkoku*.

[CR7] National Information Center of Stress and Disaster Mental Health (NICSDMH). (2026). *Koshukai ・Semina / Sutoresu・Saigaiji Kokoro no Joho Shien Senta*. https://saigai-kokoro.ncnp.go.jp/workshop.php?@rst=all

[CR32] Oliver-Smith, A., & Hoffman, S. (1999). *The angry earth: Disaster in anthropological perspective*. Routledge.

[CR34] Seale-Feldman, A. (2020). The work of disaster: Building back otherwise in post-earthquake Nepal. *Cultural Anthropology,**35*(2), 237–263. 10.14506/ca35.2.07

[CR41] Shimazono, S., & Goto, H., & Sugita, A. (2016). *Kagaku Fushin no Jidai o Tou: Fukushima Genpatsu Saigai go no Kagaku to Shakai*. Godo Shuppan.

[CR36] Starr, P. (1982). *The social transformation of American medicine*. Basic Books.

[CR37] Wynne, B. (1996). Misunderstood misunderstandings: Social identities and public uptake of science. *Misunderstanding science?: The public reconstruction of science and technology* (pp. 19–46). Cambridge University Press.

[CR38] Wynne, B. (2006). Public engagement as a means of restoring public trust in science—Hitting the notes, but missing the music? *Community Genetics,**9*(3), 211–220. 10.1159/000092659 16741352

